# Autoimmune Responses in the Rheumatoid Synovium

**DOI:** 10.1371/journal.pmed.1000009

**Published:** 2009-01-13

**Authors:** Rene E. M Toes, Tom W. J Huizinga

## Abstract

Rene Toes and Tom Huizinga discuss a new study indicating that lymphoneogenesis in the inflamed synovial tissue of patients with rheumatoid arthritis is fostering potentially pathogenic immune responses.

Rheumatoid arthritis (RA) is a chronic inflammatory disorder affecting joints, often with severe and disabling consequences. Although cells and intracellular signals characterizing inflamed tissue in RA are not qualitatively different from those found in other conditions, in about one third of patients with RA ectopic lymphoid structures can be found in affected tissue [1]. These structures, which form in response to sustained local inflammation, reflect the anatomical organization through which lymph nodes regulate the initiation and maturation of productive adaptive immune responses. Ectopic lymphoid structures therefore appear potentially capable of similarly mediating the encounter and interaction of immune cells with antigens.

## Ectopic Lymphoid Structures and Autoantibodies

It has long been speculated that ectopic lymphoid structures are not only generated in response to inflammation, but might also contribute to inflammation itself by supporting the formation and perpetuation of pathogenic immune responses [2].

Anti-citrullinated protein/peptide antibodies (ACPAs) are antibodies that recognize post-translationally modified proteins in which arginine residues have been modified into citrulline [3,4]. These antibodies are highly specific for RA, directed against antigens that are also expressed in the inflamed joint and can sometimes be detected up to ten years before disease development [5,6]. Because of these features, combined with the observations that their presence predicts clinical outcome in RA, and that their infusion exacerbates arthritis in animals, it is thought that ACPAs contribute to RA pathogenesis [7–9]. It has also been shown that synovial tissue can harbor cells that produce ACPAs [10,11].

**Linked Research Article**

This Perspective discusses the following new study published in *PLoS Medicine:*

Humby F, Bombardieri M, Manzo A, Kelly S, Blades MC, et al. (2009) Ectopic lymphoid structures support ongoing production of class-switched autoantibodies in rheumatoid synovium. PLoS Med 6(1): e1. doi:10.1371/journal.pmed.0060001

Costantino Pitzalis and colleagues show that lymphoid structures in synovial tissue of patients with rheumatoid arthritis support production of anti-citrullinated peptide antibodies, which continues following transplantation into SCID mice.

In a new study published in *PLoS Medicine* [12], Costantino Pitzalis and co-workers therefore investigated whether ectopic lymphoid structures present in the inflamed synovium of patients with RA actively contribute to ongoing B cell responses and whether they are involved in the production of ACPAs [10].

## Study Results

The authors analyzed the presence of follicular dendritic cells (FDCs) as a measure of the presence and extent of ectopic lymphoid structures. In lymph nodes, FDCs make intimate contact with B cells and play a key role in selecting antigen-binding B cells during the development of antibody responses. The authors observed that the presence of FDCs was strictly correlated with the expression of activation-induced cytidine deaminase (AID), an enzyme involved in antibody-isotype switching and affinity maturation, two processes crucial to the development of B cell antibody responses. Likewise, AID expression was correlated with markers implicated in the formation of lymphoid structures. Interestingly, cellular aggregates containing FDCs and expressing AID were found to be surrounded with cells recognizing citrullinated proteins but not control proteins, indicating the presence of ACPA-producing B cells. Together, these results indicate that the cellular aggregates associated with FDCs are functional and suggest that they contribute to the production of ACPAs.

Next the authors transplanted pieces of inflamed synovium from a series of joint biopsies in patients with RA into immunodeficient mice and followed the maintenance of germinal center–like structures in vivo as well as the survival and function of autoantibody-producing B cells. These experiments further confirmed the presence of active and self-sustained lymphoneogenesis within the inflamed synovium of patients with RA. In mice as in ex vivo human specimens, these ectopic lymphoid structures were associated with ongoing B cell responses as measured by the expression of AID and the presence of DNA recombination products that are only produced by B cells during antibody-isotype switching. ACPAs in serum were also detected, but only in those mice that were transplanted with synovial tissue that contained AID-positive cells and thus allowed for an ongoing B cell response possibly involving ACPA-isotype switching and affinity maturation.

## Strengths, Limitations, and Next Steps

The data presented by Pitzalis and co-workers show that B cells that are associated with ectopic lymphoid structures express the molecular machinery required for antibody-isotype switching and affinity maturation. Likewise, they indicate that these ectopic lymphoid structures could act as a functional tertiary lymphoid organ capable of producing isotype-switched autoantibodies. These observations are in line with the evidence suggesting that new B cells are continuously recruited into the ACPA response and indicate that the inflamed synovium is facilitating and contributing to a perpetual reactivation of the RA-specific ACPA response during the course of ACPA-positive arthritis [13].

Nonetheless, the data presented do not provide definitive proof that ectopic lymphoid structures in RA synovium support the production of ACPAs. The authors demonstrate a clear and convincing association between the presence of ectopic structures, AID-positive cells in synovial tissue, and production of ACPAs by synovial tissue, indicating that the ectopic structures and/or AID-positive cells produce or support the production of ACPAs. Nonetheless, it is not shown that disrupting the ectopic structures inhibits the formation of ACPAs. Moreover, a recent study has indicated that synovial lymphoid neogenesis is not correlated with the level of autoantibodies in RA patients, suggesting that ectopic lymphoid structures contribute little to the ACPA levels measured in serum or synovial fluid [14]. Therefore, it is important to delineate the pathogenic impact of ectopic lymphoid structures and the contribution of lymphoid neogenesis to the overall ACPA response in future studies. This could be achieved by disruption of the ectopic lymphoid structures and/or inhibition of lymphoneogenesis by targeting molecules regulating this process using the elegant mouse model developed by the authors, which closely recapitulates several of the hallmarks taking place in humans.

## Implications

The new data presented by Pitzalis and colleagues indicate that lymphoneogenesis in the inflamed synovial tissue of patients with RA is fostering potentially pathogenic immune responses by assisting the local production of ACPAs. This could promote local inflammation, leading to a vicious circle in which more lymphogenic factors are produced, allowing a further perpetuation of the autoimmune responses that drive the ongoing disease process [9]. Overall, the data presented increase our understanding of the relevance and functional consequences of the microanatomical immunological units present in a substantial number of patients with RA and provide a rationale to target these units as a new treatment modality for RA.

## Abbreviations

ACPA: anti-citrullinated protein/peptide antibody
AID: activation-induced cytidine deaminase
FDC: follicular dendritic cell
RA: rheumatoid arthritis
